# Assessing the Impact of (Poly)phenol-Rich Foods on Cardiometabolic Risk in Postmenopausal Women: A Dietary Trial

**DOI:** 10.3390/antiox13080973

**Published:** 2024-08-09

**Authors:** Lorena Sánchez-Martínez, Rocío González-Barrio, Javier García-Alonso, Pedro Mena, María-Jesús Periago

**Affiliations:** 1 Department of Food Technology, Food Science and Nutrition, University of Murcia, CEIR Campus Mare Nostrum, Campus de Espinardo, 30100 Murcia, Spain; lorena.sanchez14@um.es (L.S.-M.); fjgarcia@um.es (J.G.-A.); 2 Biomedical Reserach Institute of Murcia (IMIB-Arrixaca-UMU), University Clinical Hospital “Virgen de la Arrixaca”, El Palmar, 30120 Murcia, Spain; 3 Department of Food and Drug, University of Parma, Via Volturno 39, 43125 Parma, Italy; pedromiguel.menaparreno@unipr.it; 4 Microbiome Research Hub, University of Parma, Parco Area delle Scienze 11/A, 43124 Parma, Italy

**Keywords:** cardiovascular diseases, bioactive compounds, hypertension, metabolic health, women’s health, dark chocolate, green tea, pomegranate, berries, oranges

## Abstract

Menopause is a critical stage in a woman’s life in which cardiometabolic alterations appear, such as insulin resistance or a predisposition to visceral fat deposits, leading to an increased risk of cardiometabolic diseases (R-CMBs). New strategies to reduce the R-CMBs in postmenopausal women using natural compounds without adverse effects are desirable. In this sense, plant-based diets rich in fruits and vegetables could play a fundamental role due to the high content of bioactive compounds found in these diets, such as (poly)phenols, known for their antioxidant, anti-inflammatory and vasodilator properties. The aim of this research was to carry out a dietary trial to evaluate the effect of the daily intake of different (poly)phenol-rich foods (PP-rich foods) for 2 months on the modulation of the main cardiometabolic risk biomarkers of postmenopausal women. The results showed a slight improvement in blood pressure (BP), lipid profile and oxidative stress, endothelial function and inflammatory biomarkers. These findings suggest that daily consumption of PP-rich foods alleviated the R-CMBs of postmenopausal women by reducing the oxidative stress and, thus, the risk of cardiovascular events; however, the magnitude of the cardioprotective effect of (poly)phenols depends on inter-individual variability.

## 1. Introduction

Cardiovascular diseases (CVDs) are the main cause of death worldwide and have been described as the main cause of morbidity and mortality in women due to intrinsic factors in their physiology, such as the hormonal transition toward menopause [1]. Menopause is a critical stage in a woman’s life in which cardiometabolic alterations appear due to a sudden decline in estrogen levels and an increase in follicle-stimulating hormone and testosterone levels [2,3]. Estrogens play a cardioprotective role in the body in that they control several metabolic pathways, such as lipid metabolism and glucose metabolism, and regulate BP due to their vasodilatory effect on endothelial cells, vascular smooth muscle cells and fibroblasts [4,5,6]. Therefore, postmenopausal women experience several metabolic alterations such as dyslipidemias, insulin resistance, changes in body composition with a predisposition to visceral fat accumulation and tendency to obesity and adverse changes in vascular structure and endothelial function, leading to an increased R-CMBs. This entails the risk of different pathologies, such as metabolic syndrome, non-alcoholic fatty liver and CVDs [7,8]. Parallelly, an increase in inflammatory biomarkers and a decline in anti-inflammatory adipokines, together with an increase in reactive oxygen species (ROS) production, worsen the R-CMBs of postmenopausal women [2,9].

For this reason, several pharmacological therapies have been developed to reduce the adverse effects associated with menopause, such as hormone replacement therapy (HRT), which has been the most widely used therapy to date [10]. However, some recent prospective studies have shown a direct correlation between the use of these therapies and an increased prevalence of other pathologies, such as cancer or Alzheimer’s disease [11]. In addition, the first results from the Women’s Health Initiative study showed that HRT does not confer cardioprotective effects in all women and, in fact, may increase the risk of stroke and venous thrombosis in generally healthy older postmenopausal women [4]. Therefore, new therapies and strategies to reduce the R-CMBs in postmenopausal women with few or no adverse effects are desirable. In this sense, plant-based diets rich in fruits and vegetables could play a fundamental role in reducing the R-CMBs due to the high contents of bioactive compounds found in these diets, such as (poly)phenols [12], which are secondary metabolites widely distributed in plant foods and that have antioxidant, anti-inflammatory, vasodilator and immune response-regulating properties [13,14].

Dark chocolate, green tea, pomegranates, oranges and berries are considered suitable dietary sources of (poly)phenols [15]. Dark chocolate is known for its high flavan-3-ols content, both in the form of monomers, (+)-catechin and (−)-epicatechin, and in the form of polymers, among which procyanidin B2 stands out [16]. Green tea beverages also have a high content of flavan-3-ols, mainly (−)-epigallocatechin-3-*O*-gallate, (–)-epigallocatechin and (−)-epicatechin-3-*O*-gallate [17]. On the other hand, pomegranate and its derivatives have a high content of ellagic acid, ellagitannins, mainly punicalagin and punicalin, and several anthocyanins, such as cyanidin, pelargonidin and delphinidin [18]. Oranges provide flavanones to the diet, among which hesperidin and naringin are the most important [19], whereas berries have a high content of anthocyanins, ellagitannins and phenolic acids (hydroxycinnamic and hydroxybenzoic acids) but their (poly)phenol profile depends on the kind of berries and their varieties [20,21]. For example, raspberries are rich in cyanidin-3-*O*-sophoroside, cyanidin-3-*O*-(2″-*O*-glucosyl)rutinoside and cyanidin-3-*O*-glucoside; blueberries are rich in malvidin-3-*O*-galactoside, delphindin-3-*O*-galactoside and cyanidin-3-*O*-glucoside, and strawberries are rich mainly in pelargonidin-3-glucoside [22,23].

There is sufficient evidence supporting the positive association between the intake of these PP-rich foods and improved human health and reduced risk of chronic diseases such as cancer, CVDs and neurodegenerative diseases [14,24]. In this regard, the intake of cocoa and cocoa-rich products is associated with an improved lipid profile relating to an increase in HDL-C and a decrease in LDL-C due to the antioxidant activity of flavan-3-ols, which reduces cellular lipotoxicity [25]. Similarly, orange juice intake is associated with the control of BP due to the ability of flavanones to decrease the expression of the main hypertension-related genes [26]. Pomegranate juice consumption is associated with improved type II diabetes mellitus (T2DM) via reduced postprandial glycemic response and the induction of the expression of antioxidant enzymes, such as paraoxonase-1 [27]. In addition, green tea consumption has been shown to improve body composition and glucose metabolism due to the activity of the proanthocyanidins found in green tea, which are able to inhibit pancreatic lipase activity and, thus, suppress intestinal absorption of lipids and sugars [28]. Moreover, a decline in vascular cell adhesion molecule (sVCAM-1) and inflammatory biomarkers such as tumor necrosis factor-α (TNF-α) was observed after strawberry intake, which was associated with increased serum antioxidant capacity due to anthocyanins improving the systemic antioxidant and inflammation status [29]. Although PP-rich foods could modulate the pathophysiological alterations during menopause [30,31], a systematic review that examined the evidence of the impact of (poly)phenols on R-CMBs during the menopause showed that there are very few dietary trials in the scientific literature, and the results are sometimes contradictory [32]. Therefore, the aim of this study was to carry out a dietary trial with postmenopausal women in order to evaluate the effect of the daily intake of PP-rich foods for 2 months on the modulation of the main R-CMBs biomarkers associated with this life period. 

## 2. Materials and Methods

### 2.1. Dietary Trial

#### 2.1.1. Study Participants and Recruitment Period

**Figure 1:**
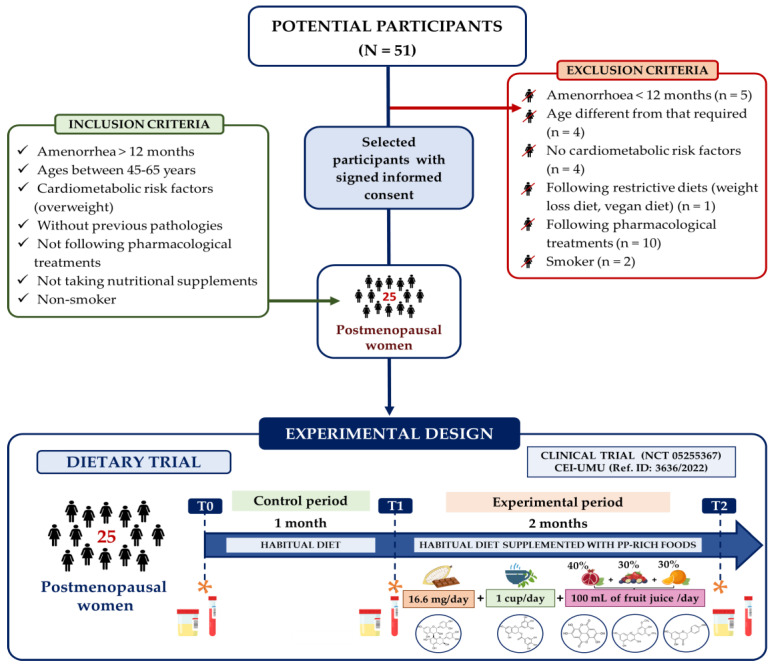
Flowchart of the recruitment period and experimental design. R-CMBs: risk of cardiometabolic diseases. Time points: (T0) baseline; (T1) control period in which participants followed their habitual diet for 1 month; (T2) experimental period, in which the diet of the participants was supplemented with (poly)phenol-rich foods for 2 months. Blood and 24 h urine were collected at T0, T1 and T2 to determine different parameters related to cardiometabolic risk, as indicated by (*).

Postmenopausal women were recruited into the study from November 2021 to April 2022. Recruitment was carried out via email, poster and information sessions in Murcia, southern Spain. A flowchart of the recruitment period is shown in Figure 1. Potential participants were screened according to the defined inclusion and exclusion criteria. The inclusion criteria included postmenopausal women 45–65 years old with at least 12 months of amenorrhea, with overweight or obesity (BMI > 25 kg/m^2^) and at least one parameter relating to R-CMBs, such as a high body fat percentage (>30%) or a high waist-to-hip ratio (WHt) (>0.85). Regarding the exclusion criteria, postmenopausal women with endocrine, hepatic or other pathologies, taking medication or dietary supplements, following a restrictive diet, allergic to any foods in the supplementation and smokers were excluded. We managed to identify 51 potential participants; however, during the selection period, 26 of them were not included as they presented one or more of the exclusion criteria. 

#### 2.1.2. Study Design

A 3-month dietary intervention was carried out, with a control period of 1 month during which the participants followed their usual diet and an experimental period of 2 months during which the participants’ diet was supplemented daily with 16.6 g of 85% cocoa dark chocolate, one cup of green tea (1 sachet in ~200 mL of boiled water) and 100 mL of fruit juice prepared from commercially refrigerated pomegranate juice (40%), orange juice (30%) and berry juice containing red grape and currants (30%). These PP-rich foods were chosen with the aim of including most of the (poly)phenol families in the diet due to their high content of proanthocyanidins (monomers and polymers), ellagitannins, flavanones and anthocyanins. However, isoflavones have not been included because the results obtained when supplementing the diet of European postmenopausal women with this type of bioactive compound are contradictory. In addition, we must emphasize that the PP-rich foods were ingested at different times (morning, afternoon and evening) with the aim of maintaining high physiological concentrations of (poly)phenols in the plasma throughout the day. During the development of the dietary trial, participants were asked to maintain their lifestyle and dietary habits. In addition, adherence to the study was followed up through a personal interview with the participants, and the nutritional composition of the diet was evaluated by completing a 24 h dietary recall. Subsequently, these data were processed using the Nutrium^®^ software v.2019 and Phenol-Explorer 3.6 database. Adherence to the Mediterranean diet and the quality of the diet were also evaluated using the questionnaire found in the PREDIMED study [33]. 

At the baseline (T0), the beginning (T1) and the end of dietary supplementation (T2), blood and 24 h urine were collected, as well as anthropometric and BP measurements. Urine samples were divided into aliquots and stored at −80 °C until analysis, while the blood samples were centrifuged at 2127× *g* for 10 min at 4 °C to obtain the plasma and then divided into aliquots and stored at −80 °C until analysis.

#### 2.1.3. Ethical Considerations

The study protocol was approved by the Research Ethics Committee of the University of Murcia (CEI:ID:3636/2022) and was registered as a clinical research study on ClinicalTrials.gov (http://www.clinicaltrials.gov (accessed on 2 August 2024), U.S. National Library of Medicine, Bethesda, MD, USA) with the accession number NCT 05255367. This study was conducted in compliance with the Declaration of Helsinki. All participants were informed about the study procedures, agreed to adhere to the stated conditions of the study and signed an informed consent form before entering the study.

### 2.2. Qualitative and Quantitative (Poly)phenol Profiles of the Foods under Study

The foods under study (85% cocoa dark chocolate, green tea and a fruit juice made of pomegranate, orange and berries) were analyzed using UHPLC-LIT-MS^n^. We followed the extraction method described by González-Barrio et al. [16,34] and the analytical method of Bresciani et al. [35], with some modifications to adapt them to the different food matrices. Briefly, 1 mL of each juice was centrifuged at 34,026× *g* for 10 min at 25 °C, the supernatants were collected, and the pellets were mixed with 500 µL of aqueous methanol acidified with formic acid (79:20:1, *v*/*v*/*v*) for pomegranate and berry juices, while 500 µL of DMSO was used for orange juice. Samples were vortexed for 1 min, sonicated for 10 min and centrifuged again under the same conditions, and the supernatants were collected. Both supernatants were mixed, and 1 mL was diluted with the same solvent of the extraction (1:20, *v*/*v*). Regarding green tea, 1 mL was centrifuged at 34,026 *g* for 10 min at 25 °C, the supernatant was collected and an aliquot of 1 mL was diluted (1:10, *v*/*v*) with aqueous methanol acidified with formic acid (79:20:1, *v*/*v*/*v*). For the 85% dark chocolate, fat was removed via the Soxhlet method, and 1 g of defatted dark chocolate was mixed with 100 mL of acetone and water (70:30, *v*/*v*), extracted for 2 h by refluxing and filtered using a Sep-pak C18 filter (500 mg, Waters) and resuspended with 3 mL of methanol [16]. After that, an aliquot of 1 mL was diluted (1:20, *v*/*v*) with aqueous methanol acidified with formic acid (79:20:1, *v*/*v*/*v*). Then, the different extracts were analyzed using UHPLC-LIT-MS (LTQ XL, Thermo Fisher Scientific Inc., San José, CA, USA) fitted with a heated ESI probe (H-ESI-II, Thermo Fisher Scientific Inc.) to identify the phenolic compounds. Separation was performed on an Acquity HSS T3 column (100 × 2.1 mm; 1.8 μm particle size; Waters, Ireland) installed with a precolumn cartridge (Waters) and maintained at 40 °C. The flow rate was set at 0.4 mL/min, the injection volume was 5 µL, and the mobile phase consisted of a mixture of acidified acetonitrile (0.1% formic acid) (solvent A) and 0.1% aqueous formic acid (solvent B). After 0.5 min of 1% solvent A in B, the proportion of A was increased linearly to 80% over a period of 9 min and maintained for 1.5 min; then, the starting conditions were re-established in 0.5 min and maintained for 2 min to re-equilibrate the column (total run time: 13 min).

Most of the native phenolic compounds in the foods were analyzed in a negative ionization mode, except for anthocyanins, which were detected in a positive ionization mode. For negative mode analysis, the H-ESI-II interface was set to a capillary temperature of 275 °C, while the source heater temperature was 200 °C. The sheath gas (N_2_) flow rate was set to 40 (arbitrary units) and the auxiliary gas (N_2_) flow rate was set to 5 (a.u.). The source voltage was 4 kV, the capillary voltage was −42 V and the tube lens voltage was −118 V. For positive mode analysis, the H-ESI-II interface was set to a capillary temperature of 275 °C and the source heater temperature was 300 °C. The sheath gas (N_2_) and auxiliary gas (N_2_) flow rates were set at 40 and 5 a.u., respectively. The source voltage was 4.5 kV, the capillary voltage was +20 and the tube lens voltage was +95 V. For both analysis negative and positive applied MS methods, ultra-pure helium gas (99.99%) was used, and a collision-induced dissociation (CID) energy equal to 35 (arbitrary units) was applied to obtain MS^2^ fragmentation. Chromatograms and mass spectral data were acquired using Xcalibur software 2.1 (Thermo Fisher Scientific Inc., Waltham, MA, USA).

### 2.3. Oxygen Radical Absorbing Capacity of the Foods

The oxygen radical absorbing capacity (ORAC) of the PP-rich foods was measured following the procedure described by Prior et al. [36]. This method is based on the inhibition of peroxy-radical-induced oxidation initiated via the thermal-based decomposition of azo compounds such as 2,2′-Azobis-(2-amidinopropane)-dihydrochloride (AAPH), using Trolox as a standard and fluorescein as a fluorescent probe. The assay was carried out in 96-well black microplates using a fluorescent microplate reader (Synergy 2 Multi-Mode Microplate Reader, Biotek, Winooski, VT, USA). For the colorimetric reaction, the samples were diluted with MilliQ water between 1:250 and 1:1000 (*v*/*v*), depending on the food under study, and an aliquot of 20 μL was mixed with 200 μL of fluorescein and pre-incubated at 37 °C for 15 min before the addition of 20 μL of AAPH solution. The fluorescence at an excitation wavelength of 485 nm and an emission wavelength of 520 nm of each well was measured every 60 s for 90 min. A Trolox calibration curve was plotted for quantification, and the obtained results were expressed as mM of Trolox equivalents.

### 2.4. Anthropometric and Blood Pressure Measurements

At the baseline and at the end of each period of the dietary intervention, anthropometric and body composition measurements were taken using a bioelectrical bioimpedance (Tanita MC-780-P), and waist and hip measurements were taken with a tape measure to determine the waist/hip (W/H) and WHt ratios. Diastolic and systolic blood pressure (DBP and SBP) and heart rate (HR) were measured in resting in duplicate using a validated semiautomatic blood pressure monitor (Omron MX2).

### 2.5. Blood Analysis, Biochemical Parameters and Antioxidant Capacity of Plasma

Blood cell counts and the biochemical analyses of plasma were carried out in an accredited clinical laboratory (Laboratorios Munuera, Murcia, Spain), including lipid profile parameters (total cholesterol, LDL-C, HDL-C, VLDL-C and triglycerides), glucose metabolism parameters (basal insulin and glucose), liver enzymes (AST-GOT and ALT-GPT) and uric acid. Furthermore, the atherogenic index of plasma (AIP), height-corrected lipid accumulation product (HLAP), triglyceride–glucose index (TyG), visceral adiposity index (VAI) [37,38], HOMA-IR index [39] and the systemic inflammatory response index (SIRI) [40] were also calculated. The ORAC of the plasma samples was also analyzed using the method previously described [36].

### 2.6. Cardiovascular Risk Biomarkers

The cardiovascular risk biomarkers analyzed in this study were as follows: oxidized LDL (Ox-LDL) related to oxidative stress status; TNF-α, adiponectin and Interleukina-6 (IL-6) as inflammatory biomarkers; and sVCAM-1 and intercellular adhesion molecules-1 (sICAM-1) as cell adhesion biomarkers. These biomarkers were measured at the beginning and at the end of the dietary trial using an enzyme-linked immunosorbent assay (ELISA) using commercially available ELISA kits for each biomarker (Elabscience). For the different analyses, the plasma was diluted with MilliQ water as required [1:2 (*v*/*v*) for Ox-LDL; no dilution for TNF-α and IL-6; 1:1500 (*v*/*v*) for adiponectin; 1:5 (*v*/*v*) for sVCAM-1; 1:20 (*v*/*v*) for sICAM-1]. All samples were assayed in duplicate according to the manufacturer’s instructions. The concentration of each molecule in the plasma was calculated using standard curves generated with the corresponding recombinant molecule.

### 2.7. Determination of Thiobarbituric Acid-Reacting Substances in Urine

The determination of thiobarbituric-acid-reacting substances (TBAR_S_) in the urine was carried out following the colorimetric method described by Buege and Aust. [41]. It is based on the reaction between lipid peroxidation products and thiobarbituric acid (TBA), which generates red abductions whose intensity can be quantified by measuring their absorbance at 532 nm. For the analysis, aliquots of 100 µL of urine samples were homogenized with 1000 µL of 0.67% TBA and 500 µL of 20% trichloroacetic acid, and the mix was incubated at 100 °C for 20 min. Then, the mix was centrifugated at 4340× *g* for 10 min, and the absorbance of the supernatants was measured at 532 nm. The total content of aldehydes capable of reacting with TBA was estimated using a molar absorption coefficient for the malondialdehyde–TBA complex of 1.56 × 10^5^ M^−1^·cm^−1^ [41].

### 2.8. Statistical Analysis

The chemical parameters of the foods were analyzed in triplicate and expressed as mean values ± standard deviation (SD). For the variables analyzed in the biological samples, the number of replications was different depending on the analysis. In particular, the biochemical analyses of plasma and the anthropometric and BP measurements were taken in duplicate, whereas ELISA kits were developed in triplicate. All these results were expressed as mean values ± standard error (SE).

The statistical analyses were performed using the SPSS statistical package (version 25, SPSS, Inc., Chicago, IL, USA). The Shapiro–Wilk test was used to verify the normal distribution of the data. T-test for dependent samples and its corresponding non-parametric Wilcoxon were applied to detect significant differences between the mean values of the parameters obtained at the beginning (T1) and at the end (T2) of the PP-rich foods supplementation period. A *p* value of <0.05 was considered statistically significant. Effect sizes were calculated using Cohen’s D, which calculates the standardized mean difference between 2 groups by subtracting the mean of one group from the mean of the other group (M1–M2) and dividing the result by the pooled SD. Effect sizes were interpreted as small (≥0.20), medium (≥0.50) and large (≥0.80) [42,43]. Effect sizes, together with the *p* value, were used to assess the magnitude of the effects observed.

## 3. Results and Discussion

### 3.1. Identification and Quantification of (Poly)phenols in Foods and Their Antioxidant Capacity

**Figure 2:**
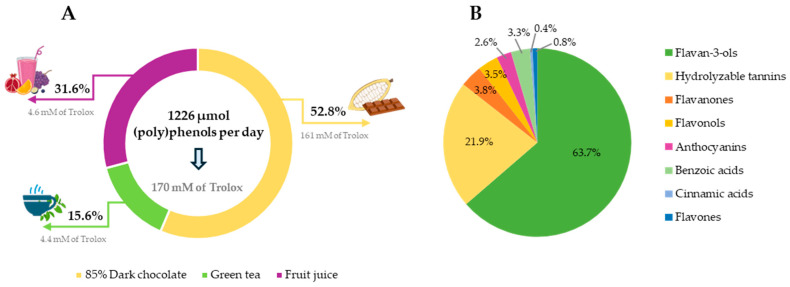
(Poly)phenolic profile of dietary supplementation. (**A**) Percentage of (poly)phenols and antioxidant capacity provided by each food included in the supplementation. (**B**) Percentage of the main (poly)phenol families provided by dietary supplementation.

Dietary supplementation with PP-rich foods, including 16.6 g of 85% cocoa dark chocolate, 200 mL of green tea and 100 mL of fruit juice, provided a total of 1226 µmol of (poly)phenols per day to the diet of postmenopausal women, as shown in Figure 2A. The identification and quantification of (poly)phenolic compounds in the foods under study are included in Table S1. Dark chocolate contributed to 52.8% of the (poly)phenol supplementation, providing 647 µmol daily, with flavan-3-ols being predominant, specifically procyanidin dimers. Fruit juice followed, which contributed 31.6% of the (poly)phenol supplementation, providing 387 µmol daily. It was mainly composed of pomegranate ellagitannins (punicalin and punicalagin) and ellagic acid derivatives, anthocyanins, such as cyanidin-3,5-diglucoside, and orange flavanones, with hesperidin and naringenin being predominant. The daily intake of green tea contributed 15.6% of the (poly)phenol supplementation, providing 192 µmol daily, and (epi)gallocatechin-3-*O*-gallates were the predominant green tea flavan-3-ols. 

Figure 2B shows the (poly)phenol profile of the supplementation by families, which was represented by flavan-3-ols (63.7%), hydrolyzable tannins (21.9%), flavanones (3.8%), flavonols (3.5%), anthocyanins (2.6%), phenolic acids (3.7%) (hydroxycinnamic and hydroxybenzoic acids) and flavones (0.8%). It should be noted that the selected foods have a great ORAC due to their content of (poly)phenols, and their intake significantly improved the total antioxidant capacity of the supplemented diet (providing a mean value of 170 mM of Trolox). Regarding the content of bioactive compounds, dark chocolate was the food that contributed the greatest antioxidant capacity found in the diet, with an average content of 161 mM of Trolox, followed by fruit juice with an average value of 4.6 mM of Trolox (pomegranate juice provided 3.1 mM of Trolox; berry juice provided 0.95 mM of Trolox; and orange juice provided 0.63 mM of Trolox) and by green tea, with an average value of 4.4 mM of Trolox (Figure 2A).

### 3.2. Participant Characteristics at Baseline

The baseline characteristics of the participants included in this dietary trial are summarized in Table 1. The age of the postmenopausal women was 56.16 years. Baseline anthropometric measurements revealed that most participants were overweight (64%) or obese (36%) according to their BMI (with a mean value of 29.06 kg/m^2^) and their W/H ratio (with a mean value of 0.89), following the classification of the WHO European Regional Obesity Report [44]. Furthermore, the body fat percentage of the participants also ranged from overweight (30.20%) to obese (44.40%).

BP measurements showed that most of the participants were normotensive, with a mean SBP value of 129 mmHg and a mean DBP value of 83.50 mmHg [45,46]. On the other hand, biochemical measurements of glucose metabolism were within desirable levels, with a mean fasting glucose value of 92.72 mg/dL, a mean fasting insulin value of 9.73 μUI/mL and a mean HOMA-IR index value of 2.14 [47]. However, the participants presented high levels of total cholesterol and LDL-C with a mean value of 232.04 mg/dL and 145.04 mg/dL, respectively [48]. The participants presented a medium risk of adverse cardiovascular events, with a mean AIP value of 0.13 [37].

Related to the dietary history of the postmenopausal women enrolled in the dietary intervention trial (Table 1), it should be noted that most of the participants showed a high adherence to the Mediterranean diet, with a mean Med-Score value of 9.27, indicating healthy eating habits from a qualitative point of view. The intake of (poly)phenols was slightly higher than the average intake of the Spanish population, which is 1360 mg/person/day [49].

In terms of energy intake and macronutrient distribution, the diet provided an average value close to 1900 kcal/day, of which 42.72% was from carbohydrates, 19.33% from proteins and 37.69% from fats. Like the European population and, in particular, the Spanish population, the participants’ diet was characterized by a high intake of proteins and lipids to the detriment of carbohydrates [50,51], which could lead to an increased R-CMBs, particularly due to the high fat intake. 

### 3.3. Dietary Intervention Effect on Anthropometric and Blood Pressure Measurements, Lipid Profile and Glucose Homeostasis Indicators

Table 2 includes the mean values of these parameters at the beginning (T1) and after 2 months of daily supplementation with PP-rich foods (T2) and the value of Cohen’s D used to describe the effect size and the participants in which changes were observed. Most of the parameters decreased in around 50% or more of the participants, but the mean values did not show significant differences due to the inter-individual variability among the participants.

**Figure 3:**
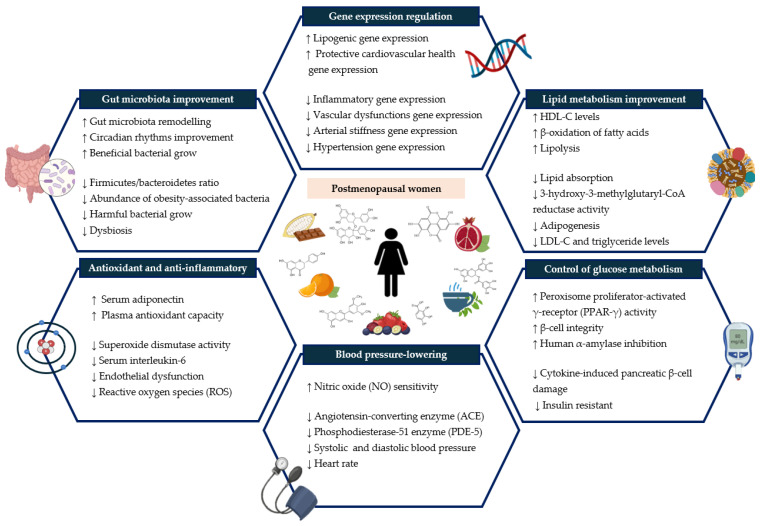
Potential mechanisms of (poly)phenol-rich foods to improve cardiometabolic and cardiovascular risk in postmenopausal women. ↑: Variables that increase; ↓: Variables that decrease.

Regarding anthropometric measurements, it is known that postmenopausal women experience changes in body composition and, particularly, gain about 0.68 kg per year, and they have 49% more abdominal fat compared to premenopausal women. This factor increases the R-CMBs and the development of T2DM [52]. However, this risk could be reduced via the potential anti-obesity mechanisms of (poly)phenols (Figure 3). In this study, slight modulations of anthropometric measurements (i.e., BMI and body weight) were observed, which may be associated with the ability of proanthocyanidins found in green tea to inhibit pancreatic lipase activity, thus reducing intestinal lipid absorption and, therefore, the total energy intake [28]. However, no significant differences were observed, as previously described in other studies, particularly after the intake of tea extract for 2 months [53] and after the consumption of 10 g of cocoa-rich chocolate for 6 months [54].

BP was also measured as one of the major risk factors for CVDs, which increases in postmenopausal women due to the decreased cardioprotective effect of estrogens via their control of blood vessels. This also ought to decrease the synthesis of nitric oxide (NO) [4,55]. In this context, at the beginning of the experimental period (T1), the participants showed high BP levels, but after the experimental period, a moderate effect size on both SBP and DBP was observed, with Cohen’s D values of 0.261 and 0.242, respectively. SBP decreased from a mean value of 130.80 mmHg at T1 to a mean value of 126.24 mmHg at T2, whereas DBP decreased from 84.52 to 82.16 mmHg. It should be noted that this reduction could be considered relevant from a clinical point of view since each increase of 2 mmHg in SBP increases mortality from ischemic heart disease and stroke by 7% and 10%, respectively [56]. This finding agrees with the scientific literature since the intake of dark chocolate (10 g during 6 months) decreased SBP and DBP in postmenopausal women [54]. In fact, a recent meta-analysis suggests that the consumption of more than 2.5 g of dark chocolate per day is associated with a significant reduction in BP in the general population, independent of age, BMI and/or study duration [25]. This effect could be due to the daily intake of cocoa flavan-3-ols, which stimulate the production of NO in endothelial cells via NO synthase, exerting a vasodilatory effect that lowers BP; moreover, it is also antiatherogenic and reduces cellular inflammation and platelet activation (Figure 3) [25,57]. 

During menopause, the occurrence of overweight, the tendency to develop obesity and the redistribution of body fat into the visceral area lead to increased insulin resistance [5,55]. In this context, the HOMA-IR index has been proven to identify the risk of insulin resistance in the general population and predict the development of adverse effects associated with this metabolic alteration [58]. At the beginning of the experimental period (T1), the participants showed a HOMA-IR index of 2.62, indicating a suspected risk of insulin resistance [39,58], but after the diet supplementation with PP-rich foods (T2), this index decreased slightly, reaching a mean value of 2.57, which is associated with a decrease in fasting glucose (93.88 vs. 92.40 mg/dL). In this intervention study, both cocoa flavan-3-ols and pomegranate ellagitannins and ellagic acid could have contributed to modulating glucose metabolism. This is because they are recognized as being able to maintain glucose homeostasis by antagonizing digestive enzymes and glucose transporters, as well as modulating insulin secretion in pancreatic cells by regulating key proteins in the insulin signaling pathway, thus contributing to the prevention of T2DM [25,59]. However, previous studies have shown a high inter-individual variability regarding the hypoglycemic effect of (poly)phenols in the general population [60] and in postmenopausal women [61,62], showing that the hypoglycemic capacity of (poly)phenols is strongly linked to the physiological state of the individual. For example, Ramos-Romero et al. [60] showed that in states of insulin resistance, (poly)phenols are able to act on glucose metabolism and significantly reduce the risk of developing diabetes. On the contrary, under physiological conditions of normoglycemia, (poly)phenols act by maintaining the state of homeostasis, and no significant differences are usually seen after supplementing the diet of these populations with (poly)phenols. 

In addition to the changes in glucose metabolism, dyslipidemias are also associated with menopause, increasing total cholesterol, LDL-C (10–20%) and triglycerides (10–15%), together with a decrease in HDL-C, leading to increased atherosclerotic processes and higher R-CMBs [55,63]. After the experimental period with PP-rich foods for 2 months, a moderate effect size was observed for plasmatic triglycerides and VLDL-C, with Cohen’s D values of 0.288 and 0.294, respectively. Triglycerides decreased from a mean value of 106.07 mg/dL at T1 to a mean value of 89.46 mg/dL at T2, whereas VLDL-C decreased from 23.11 to 19.27 mg/dL during the experimental period. As a result of the modification of plasmatic lipids, the cardiovascular indices, TyG, HLAP and VAI, exhibited a moderate improvement, with Cohen’s D values of 0.234, 0.284 and 0.246, respectively. However, these changes were not statistically significant due to the inter-individual variability observed [64,65]. Other authors have reported improved lipid plasmatic levels in postmenopausal women after supplementing the diet with a (poly)phenol from dried plum (for 12 months) [66] or green tea extract (for 2 months) [53]; however, no statistically significant differences were observed between the control and experimental periods, similar to our findings.

### 3.4. Dietary Intervention Effect on Inflammatory, Endothelial Function and Oxidative Stress Biomarkers and Plasma Antioxidant Capacity

During menopause, the risk of oxidative stress and chronic inflammation is also increased [6,67]. In addition, adipose tissue acts as a dynamic endocrine organ through the synthesis of adipokines, including cardioprotective adipokines, such as adiponectin, but also proinflammatory adipokines, such as TNF-α, IL-6 and plasminogen activator inhibitor-1 [68]. The increase in these proinflammatory cytokines and the decrease in cardioprotective adipokines are associated with endothelial dysfunction mediated by the increase in sVCAM-1 and sICAM-1, and this process constitutes the beginning of atherosclerosis [2,52]. On the other hand, the efflux of free fatty acids into the bloodstream associated with obesity generates a greater amount of ROS, leading to a state of oxidative stress [64]. Therefore, the intake of bioactive compounds with high anti-inflammatory and antioxidant properties, such as (poly)phenols, which can act as free radical scavengers and inhibit lipid peroxidation, as well as promote the expression of antioxidant genes through the activation of signaling pathways endogenous antioxidants (Figure 3) [24,29,69], could be a suitable strategy to reduce the oxidative and inflammatory state generated during menopause.

In this context, this work showed an improvement in inflammatory biomarkers (adiponectin and SIRI index), endothelial function biomarkers (sICAM-1 and sVCAM-1) and oxidative stress biomarkers (TBARs) in postmenopausal women after the intervention; however, only TBARs showed significant differences between T1 and T2 (Table 3). 

As regards the inflammatory biomarkers, in the plasmatic concentration of TNF-α, a slight significant increase was observed when comparing the T1 and T2 values (0.16 vs. 0.21 ng/mL). However, the moderate effect size observed for the cardioprotective cytokine adiponectin should be highlighted, with a Cohen’s D value of 0.349, reaching a mean value of 113 µg/mL after the experimental period, wherein improved levels of this biomarker in 17 out of 25 participants were observed. Related to the effect of (poly)phenols on inflammatory biomarkers, improved adiponectin levels after ingesting capsules containing cocoa and soy (poly)phenols for 6 months have been reported [70], while TNF-α did not exhibit significant differences after the intake of elderberry capsules during 3 months of intake in postmenopausal women [71]. The increase in adiponectin could contribute to improved R-CMBs since although no significant differences were observed in TNF-α and adiponectin, the former decreased in 32% of the participants, whereas the second increased in 68% of the postmenopausal women. 

Furthermore, there was a slight decline in the SIRI index in more than 50% of the participants; however, this was not statistically significant. The SIRI index is directly related to the R-CVDs, particularly the risk of death from stroke and myocardial infarction [72,73]. Previous studies have indicated that this close relationship relates to the fact that neutrophils play a fundamental role in the inflammatory response of atherosclerosis. Neutrophils can secrete a range of inflammatory mediators, chemotactic agents and ROS that induce endothelial cell injury and tissue ischemia. The activation of monocytes and their subsequent transformation into macrophages represents a pivotal process in the formation of atherosclerotic lesions. On the contrary, lymphocytes have a regulatory function in inflammation and can exert an inhibitory effect on atherosclerosis [73]. Therefore, the consumption of bioactive compounds with immunomodulatory properties, such as (poly)phenols, may be an effective strategy to reduce the SIRI index of postmenopausal women.

Regarding endothelial function biomarkers, a slight decrease in sICAM-1 and sVCAM-1 was observed, from mean values of 82.37 ng/mL and 188.50 ng/mL at T1 to mean values of 79.87 ng/mL and 169.35 ng/mL at T2, respectively. In this sense, the improvement observed in sICAM-1 and sVCAM-1 agrees with the data provided by the scientific literature. For example, Wang-Polagruto et al. [74] showed a significant decrease in sVCAM-1 in hypercholesterolemic postmenopausal women after the ingestion of a cocoa drink rich in flavan-3-ols for 6 months, which was associated with the ability of cocoa flavan-3-ols to inhibit the activation of the oxidative stress-sensitive nuclear transcription factor kappa-B, which is a promoter of sVCAM-1 expression [75]. Moreover, Estévez-Santiago et al. [61] observed a decrease in both sVCAM-1 and sICAM-1 in postmenopausal women after the intake of supplements in the form of capsules with anthocyanins for 8 months; however, this improvement was not significant.

For the oxidative stress biomarkers, improved participant oxidative stress status after diet supplementation with PP-rich foods was shown based on the decreases in Ox-LDL (from 17.21 ng/mL at T1 to 15.36 ng/mL at T2) and TBARs (from 6.81 µmol/g creatinine at T1 to 3.84 µmol/g creatinine at T2). The TBARs concentration diminished significantly from the initial levels of around 45%, which could be due to the antioxidant capacity of the flavan-3-ols in cocoa and green tea and the phenolic acids and anthocyanins present in the pomegranate and berry juices. Other studies, such as that conducted by Kardum et al. [76], also reported improved TBARs after the intake of chokeberry juice for 3 months in healthy women. Similarly, Nanetti et al. [77] showed a significant decrease in TBARs in both men and women after the intake of 50 g of flavonoid-rich dark chocolate across 3 weeks. 

These results are in line with those reported in the systematic review of the benefits of (poly)phenol consumption on R-CMBs in postmenopausal women that we recently conducted. Most of the studies evaluated showed a trend toward reduced R-CMBs biomarkers after (poly)phenol supplementation, but very few studies showed significant differences. Thus, this review concluded that the effects of (poly)phenol intake during menopause on the modulation of inflammatory, endothelial function and oxidative stress biomarkers are modest and depend on inter-individual variability [32].

## 4. Strengths and Limitations of the Study

This dietary intervention trial, designed as a single-arm study, is the first investigation to evaluate the supplementation of the diet with a wide variety of (poly)phenol families (except isoflavones) using common foods (85% cocoa dark chocolate, green tea and fruit juice) on the R-CMBs of postmenopausal women. The study did not include a control group, but a control period of 1 month was taken into consideration to evaluate the possible changes in the physiological parameters and in the different variables under study of the participants before the supplementation period to compare the experimental or supplementation period. 

The normal diet of the participants was supplemented daily for 2 months with 16.6 g of 85% cocoa dark chocolate, a cup of green tea (~200 mL) and 100 mL of fruit juice without intentional dietary modifications during the study. In addition, the participants were not blinded because we used a natural dietary source of bioactive compounds. Although no significant differences were observed between the beginning and the end of the experimental period in most of the analyzed variables, except for TBARs, in terms of R-CMBs, a slight improvement was observed in most of them. Therefore, the results of this study revealed that daily supplementation with PP-rich foods has a relevant impact on the cardiovascular health of postmenopausal women by significantly reducing the oxidative stress state through decreased TBAR levels. 

On the other hand, it should be noted that this study has some limitations due to the lack of a placebo group and the fact that during the study, the participant followed their habitual diet based on a Mediterranean dietary pattern. However, large variability was observed in adherence to the Mediterranean diet (Table 1) and, thus, in the total intake of (poly)phenols, which may have also influenced the results obtained. Moreover, our findings suggest that there is a high inter-individual variability in the metabolic responses to (poly)phenol intake and, therefore, in the expected beneficial effects that these bioactive compounds could exert in this specific population. This could be largely due to the activity of the gut microbiota, which plays a fundamental role in (poly)phenol metabolism, conditioning the type and quantity of derived metabolites and, therefore, the magnitude of the beneficial effect that these compounds exert on human health [78].

Further research we have carried out in this line includes the characterization of phase II and microbial-derived metabolites and the analysis of gut microbiota composition and its metabolic activity, as well as gene expression after the intake of PP-rich foods. These results complement those of the present work and will provide valuable information in terms of explaining the possible overall beneficial effect that (poly)phenols could bring to women’s health.

## 5. Conclusions

After 2 months of daily supplementation with PP-rich foods in postmenopausal women, a slight improvement was observed in BP (SBP and DBP), lipid profile (triglycerides, VLDL and TyG, HLAP and VAI indices), oxidative stress biomarker (TBARs), endothelial function biomarkers (sICAM-1 and sVCAM-1) and anti-inflammatory biomarkers (adiponectin). However, this improvement was only statistically significant for TBARs. Therefore, our results suggest that the daily consumption of 16.6 g of 85% cocoa dark chocolate, 200 mL of green tea and 100 mL of fruit juice improves the cardiometabolic health of postmenopausal women by reducing oxidative stress and, thus, the risk of cardiovascular events. However, the magnitude of the cardioprotective effect of (poly)phenols largely depends on inter-individual variability. Therefore, further studies evaluating the impact of other factors involved, such as the bioavailability of the (poly)phenols and inter-individual variability and the role of gut microbiota on the colonic metabolization of these bioactive compounds, as well as the modulation of the gene expression via nutrigenomic effects related to cardiometabolic and cardiovascular risks, are warranted to explain the potential benefit of these compounds in postmenopausal women.

## Figures and Tables

**Table 1:** Baseline characteristics of postmenopausal women included in the intervention study (n = 25) *.

General Characteristics	Mean ± SE	Minimum	Maximum
Anthropometric measurements			
Age (years)	56.16 ± 0.75	51.00	64.00
Height (m)	1.60 ± 0.01	1.48	1.75
Body weight (kg)	74.72 ± 1.96	59.10	98.10
BMI (kg/m^2^)	29.06 ± 0.58	25.00	35.60
Body fat (%)	36.24 ± 0.80	30.20	44.40
Body fat index	8.60 ± 0.35	6.00	12.00
W/H ratio	0.89 ± 0.01	0.73	1.00
WHt ratio	0.61 ± 0.01	0.52	0.69
Blood pressure measurements			
SBP (mmHg)	129.48 ± 0.38	103.00	174.00
DBP (mmHg)	83.50 ± 0.21	63.00	105.00
Heart rate (heartbeats/min)	73.56 ± 1.84	59.00	96.00
Biochemical measurements of glucose metabolism			
Fasting glucose (mg/dL)	92.72 ± 2.68	77.00	138.10
Fasting insulin (μUI/mL)	9.73 ± 1.29	3.00	29.59
HOMA-IR index	2.14 ± 0.39	0.00	7.43
Biochemical measurements of lipid profile			
TC (mg/dL)	232.04 ± 8.86	162.00	315.70
Triglycerides (mg/dL)	95.08 ± 8.03	46.00	225.10
HDL-C (mg/dL)	66.32 ± 2.88	45.00	99.90
LDL-C (mg/dL)	145.04 ± 7.27	89.00	226.91
VLDL-C (mg/dL)	19.34 ± 1.70	9.00	45.03
AIP	0.13 ± 0.04	−0.17	0.53
TyG index	8.30 ± 0.08	7.63	9.21
HLAP index	0.66 ± 0.06	0.29	1.64
VAI	3.07 ± 0.32	7.63	9.21
Liver enzymes and uric acid			
AST-GOT (UL/L)	20.70 ± 1.22	8.60	39.00
ALT-GPT (UL/L)	23.06 ± 1.81	10.00	47.60
Uric acids (mg/dL)	4.42 ± 0.26	2.60	8.19
Dietary history			
MedDiet (14-item score)	9.27 ± 0.46	4.00	13.00
Total PP intake (mg/day)	1891.02 ± 179.11	127.52	4331.37
Total energy intake (kcal/day)	1872.19 ± 82.63	1282.33	2881.00
Fats (%)	37.69 ± 1.03	29.67	49.05
Saturated fats (%)	41.48 ± 0.88	32.23	49.73
Proteins (%)	19.33 ± 0.54	14.70	25.67
Carbohydrates (%)	42.72 ± 0.81	34.60	49.03
Sugars (%)	33.35 ± 1.77	11.43	51.47

* Data are expressed as the mean ± standard error (SE) for continuous variables and percentage (%) for categorical variables. BMI: body mass index; W/H: waist–hip ratio; WHt: waist-to-height ratio; SBP: systolic blood pressure; DBP: diastolic blood pressure; HOMA-IR index: Homeostasis Model Assessment of Insulin Resistance; TC: total cholesterol; HDL-C: high-density lipoprotein cholesterol; LDL-C: low-density lipoprotein cholesterol; VLDL-C: very low-density lipoprotein cholesterol; AIP: atherogenic index of plasma; TyG index: triglyceride–glucose index; HLAP: height-corrected lipid accumulation product; VAI: visceral adiposity index; MedDiet: Mediterranean Diet Adherence Screener 14-item score; PP: (poly)phenols.

**Table 2:** Intervention effect during the experimental period on anthropometric and blood pressure measurements, glucose homeostasis indicators and lipid profile.

	Beginning (T1)	End (T2)			
	Mean ± SE	Mean ± SE	*p* Value	Effect Size (Cohen’s D)	N
Anthropometric measurements					
Body weight (kg) ^b^	74.68 ± 1.93	74.51 ± 1.92	0.553	0.018	↓ (15/25)
BMI (kg/m^2^) ^a^	29.02 ± 0.57	28.96 ± 0.59	0.530	0.022	↓ (15/25)
Body fat (%) ^a^	35.87 ± 0.89	35.42 ± 0.89	0.231	0.101	↓ (14/25)
Body fat index ^a^	8.52 ± 0.39	8.48 ± 0.37	0.746	0.021	↓ (05/25)
W/H ratio ^b^	0.89 ± 0.01	0.89 ± 0.01	0.721	0.065	↓ (12/25)
WHt ratio ^a^	0.61 ± 0.01	0.61 ± 0.01	0.238	0.095	↓ (12/25)
Blood pressure measurements					
SBP (mmHg) ^a^	130.80 ± 3.82	126.24 ± 3.13	0.193	0.261	↓ (14/25)
DBP (mmHg) ^a^	84.52 ± 2.03	82.16 ± 1.87	0.228	0.242	↓ (11/25)
HR (heartbeats/minute) ^a^	74.44 ± 2.41	73.44 ± 2.01	0.573	0.090	↓ (13/25)
Biochemical measurements of glucose metabolism					
Fasting glucose (mg/dL) ^b^	93.88 ± 2.15	92.40 ± 2.61	0.501	0.123	↓ (14/25)
Fasting insulin (μUI/mL) ^b^	11.08 ± 0.77	11.08 ± 0.99	0.753	0.002	↓ (12/24)
HOMA-IR index ^b^	2.62 ± 0.24	2.57 ± 0.29	0.648	0.036	↓ (13/24)
Biochemical measurements of lipid profile					
TC (mg/dL) ^a^	217.18 ± 5.71	216.26 ± 5.36	0.753	0.033	↓ (13/25)
Triglycerides (mg/dL) ^b^	106.07 ± 14.77	89.46 ± 6.87	0.206	0.288	↓ (14/25)
HDL-C (mg/dL) ^a^	61.09 ± 2.45	60.42 ± 2.44	0.531	0.054	↑ (11/25)
LDL-C (mg/dL) ^a^	134.83 ± 5.32	137.95 ± 4.93	0.397	0.122	↓ (11/25)
VLDL-C (mg/dL) ^b^	23.11 ± 3.58	19.27 ± 1.92	0.878	0.294	↓ (04/15)
AIP ^a^	0.19 ± 0.05	0.15 ± 0.04	0.274	0.150	↓ (13/25)
TyG index ^b^	8.37 ± 0.11	8.25 ± 0.08	0.135	0.234	↓ (16/25)
HLAP index ^b^	0.74 ± 0.11	0.62 ± 0.05	0.178	0.284	↓ (14/25)
VAI index ^b^	3.93 ± 0.77	3.20 ± 0.35	0.326	0.246	↓ (13/25)
Liver enzymes and uric acid					
AST-GOT (UL/L) ^a^	19.58 ± 0.26	22.04 ± 1.13	0.021 *	0.413	↓ (07/25)
ALT-GPT (UL/L) ^b^	21.56 ± 1.24	21.77 ± 1.90	0.542	0.024	↓ (12/25)
Uric acids (mg/dL) ^b^	4.41 ± 1.61	4.81 ± 0.32	0.011 *	0.272	↓ (08/25)

N: number of participants; ↓: individuals for whom the variable of interest decreases from T1 to T2; ↑: individuals for whom the variable of interest increases from T1 to T2; BMI: body mass index; W/H: waist–hip ratio; WHt: waist-to-height ratio; SBP: systolic blood pressure; DBP: diastolic blood pressure; HR: heart rate; HOMA-IR index: Homeostasis Model Assessment of Insulin Resistance; TC: total cholesterol; HDL-C: high-density lipoprotein cholesterol; LDL-C: low-density lipoprotein cholesterol; VLDL-C: very low-density lipoprotein cholesterol; AIP: atherogenic index of plasma; TyG index: triglyceride–glucose index; HLAP: height-corrected lipid accumulation product; VAI: visceral adiposity index. Effect size was interpreted as small (≥0.20), medium (≥0.50) and large (≥0.80). Data are expressed as the mean ± standard error (SE). Means within the same row with (*) are significantly different *p* value < 0.05). (^a^): normal distribution; (^b^): not normal distribution.

**Table 3:** Effect of dietary interventions on inflammatory, endothelial function and oxidative stress biomarkers.

	Beginning (T1)	End (T2)			
	Mean ± SE	Mean ± SE	*p* Value	Effect Size (Cohen’s D)	N
Inflammatory biomarkers					
TNF-α (ng/mL) ^b^	0.16 ± 0.04	0.21 ± 0.05	0.153	0.231	↓ (08/25)
Adiponectin (µg/mL) ^a^	96.78 ± 9.29	113 ± 9.32	0.083	0.349	↑ (17/25)
SIRI index ^b^	0.59 ± 0.05	0.57 ± 0.04	0.689	0.066	↓ (13/24)
Endothelial adhesion biomarkers					
sICAM-1 (ng/mL) ^b^	82.37 ± 6.06	79.87 ± 6.13	0.543	0.087	↓ (12/22)
sVCAM-1 (ng/mL) ^b^	188.50 ± 50.79	169.35 ± 48.75	0.627	0.086	↓ (07/20)
Oxidative stress biomarkers					
Ox-LDL in plasma (ng/mL) ^b^	17.21 ± 4.17	15.36 ± 2.46	0.458	0.109	↓ (09/24)
TBARs in urine (µmol/g creatinine) ^b^	6.81 ± 0.52	3.84 ± 0.28	<0.001 *	1.445	↓ (21/24)
Plasma antioxidant capacity					
ORAC (mM Trolox equivalents) ^a^	29.51 ± 0.61	28.93 ± 0.68	0.524	0.202	↑ (08/20)

N: number of participants; ↓: individuals for whom the variable of interest decreases from T1 to T2; ↑: individuals for whom the variable of interest increases from T1 to T2; TNF-α: tumor necrosis factor-α; SIRI: systemic inflammatory response index; sICAM-1: intercellular adhesion molecule; sVCAM-1: vascular cell adhesion molecule; Ox-LDL: oxidized LDL; TBARs: thiobarbituric acid-reacting substances; ORAC: oxygen radical absorbing capacity method. Effect size was interpreted as small (≥0.20), medium (≥0.50) and large (≥0.80). Data are expressed as the mean ± standard error (SE). Means within the same row with (*) are significantly different (*p* value < 0.001). (^a^): normal distribution; (^b^): not normal distribution.

## Data Availability

Data is contained within the article or Supplementary Material.

## Supplementary Materials

The following supporting information can be downloaded at https://www.mdpi.com/article/10.3390/antiox13080973/s1, Table S1: Identification and quantification of (poly)phenolic compounds in 85% cocoa dark chocolate, green tea and fruit juice ingested during the experimental period and mass spectrometric and chromatographic characteristics.
