# Supplementary material for: Assessing the Impact of (Poly)phenol-Rich Foods on Cardiometabolic Risk in Postmenopausal Women: A Dietary Trial
Source: Antioxidants (Basel). 2024 Aug 9;13(8):973. doi: 10.3390/antiox13080973 (PMC11351953; doi:10.3390/antiox13080973)
Supplement: Supplementary file 1 [file antioxidants-13-00973-s001.zip › antioxidants-3108573-supplementary.pdf]

Table S1. Identification and quantification of (poly)phenolic compounds in 85% cocoa dark chocolate, green tea, and fruit juice ingested during the experimental period, and mass spectrometric and chromatographic characteristics. Data are expressed as μmol per daily food intake (mean values ± SD, n = 3).

Compounds	RT (min)	$[M-H]^-$ or $[M]^{/+}$ (m/z)	MS^2 fragment ions (m/z)	Standard used for quantification	85% Cocoa dark chocolate	Green tea	Fruit juice	Overall Supplementation
					Daily intake (μmol)			
**Hydroxybenzoic acids**								
3,4,5-Trihydroxybenzoic acid (gallic acid) [a]	1.45	169^-	125, 107	3,4,5-Trihydroxybenzoic acid	ND	7.78 ± 0.62	17.29 ± 1.21	25.07 ± 1.83
3,4-Dihydroxybenzoic acid (protocatechuic acid) [b]	2.40	153^-	123, 109	3,4,5-Trihydroxybenzoic acid	ND	ND	4.46 ± 0.19	4.46 ± 0.19
4-Hydroxy-3-methoxybenzoic acid (vanillic acid) [b]	3.30	167^-	123,152,125	3,4,5-Trihydroxybenzoic acid	ND	ND	10.50 ± 0.66	10.50 ± 0.66
Total Hydroxybenzoic acids					**ND**	**7.78 ± 4.49**	**32.25 ± 6.42**	**40.03 ± 10.59**
**Hydroxycinnamic acids**								
cis-3',4'-Dihydroxycinnamic acid [b]	2.60	179^-	135	3-*O*-Galloylquinic acid	ND	ND	3.63 ± 0.18	3.63 ± 0.18
trans-3',4'-Dihydroxycinnamic acid [b]	3.20	179^-	121, 135, 109	3-*O*-Galloylquinic acid	ND	ND	1.78 ± 0.04	1.78 ± 0.04
Total Hydroxycinnamic acids					**ND**	**ND**	**5.41 ± 1.31**	**5.41 ± 1.31**
**Hydrolyzable tannins and ellagic acid derivatives**								
Punicalagin-like [b]	0.67	1083^-	601, 721	Punicalin α	ND	ND	5.29 ± 0.27	5.29 ± 0.27
Punicalagin α [b]	2.55	1083^-	601, 781	Punicalagin β	ND	ND	0.92 ± 0.09	0.92 ± 0.09
Punicalagin β [a]	2.72	1083^-	781, 601	Punicalagin β	ND	ND	2.28 ± 0.29	2.28 ± 0.29
Punicalin (α/β) [a]	1.64	781^-	601, 721	Punicalin α	ND	ND	19.89 ± 0.13	19.89 ± 0.13
Pedunculagin I isomer [b]	2.66	783^-	765, 301	Punicalagin β	ND	ND	8.83 ± 0.92	8.83 ± 0.92
Puniglyconin [b]	2.86	801^-	301, 347, 649	3-*O*-Galloylquinic acid	ND	ND	6.46 ± 0.78	6.46 ± 0.78
Granatin B [b]	3.19	951^-	933, 613	Procyanidin dimer (B1-type)	ND	ND	1.10 ± 0.20	1.10 ± 0.20
Galloyl-hexoside [b]	1.06	331^-	169, 271	3-*O*-Galloylquinic acid	ND	ND	10.02 ± 0.41	10.02 ± 0.41
Galloyl-HHDP-Hexoside [b]	3.00	633^-	301, 463, 275	3-*O*-Galloylquinic acid	ND	ND	7.78 ± 0.79	7.78 ± 0.79
3-*O*-Galloylquinic acid [a]	0.96	343^-	169, 191	3-*O*-Galloylquinic acid	ND	ND	0.85 ± 0.12	0.85 ± 0.12
4-*O*-Galloylquinic acid [b]	1.70	343^-	169, 191	3-*O*-Galloylquinic acid	ND	ND	ND	ND
5-*O*-Galloylquinic acid [b]	1.41	343^-	191, 196	3-*O*-Galloylquinic acid	ND	1.53 ± 0.15	ND	1.53 ± 0.15

Ellagic acid [b]	3.55	301-	257, 229	3-*O*-Galloylquinic acid	ND	ND	119.20 ± 12.70	119.20 ± 12.70
Ellagic acid-pentoside [b]	3.37	433-	301, 415, 433 447, 285	3-*O*-Galloylquinic acid	ND	3.64 ± 0.36	8.07 ± 0.67	11.72 ± 0.60
Ellagic acid-hexoside [b]	3.04	463-	301, 257, 229	3-*O*-Galloylquinic acid	ND	ND	53.33 ± 1.85	53.33 ± 1.85
Ellagic acid-deoxyhexoside [b]	3.37	447-	300, 301	3-*O*-Galloylquinic acid	ND	ND	19.46 ± 0.73	19.46 ± 0.73
Total Hydrolyzable tannins and ellagic acid derivatives					**ND**	**5.17 ± 0.96**	**263.49 ± 30.41**	**268.66 ± 30.30**
**Flavan-3-ols**								
(+)-Catechin [a]	2.92	289-	245, 205	(+)-Catechin	87.76 ± 7.66	15.83 ± 0.97	2.20 ± 0.09	105.79 ± 7.26
(–)-Epicatechin [a]	3.15	289-	245, 205	(–)-Epicatechin	221.45 ± 9.97	12.98 ± 0.59	0.33 ± 0.01	234.77 ± 9.37
Gallocatechin [b]	2.34	305-	179, 221	(–)-Epicatechin	ND	9.50 ± 0.23	0.24 ± 0.03	9.74 ± 0.22
Epigallocatechin [b]	2.75	305-	179, 221	(–)-Epicatechin	ND	15.28 ± 1.16	ND	15.28 ± 1.16
Gallocatechin-3'-*O*-gallate [b]	3.16	457-	169, 331	Epigallocatechin-3'-*O*-gallate	ND	55.34 ± 2.40	ND	55.34 ± 2.40
Epicatechin-3'-*O*-gallate [a]	3.57	441-	301, 300	Epicatechin-3'-*O*-gallate	ND	37.77 ± 2.27	ND	37.77 ± 2.27
Epigallocatechin-3'-*O*-gallate [a]	3.24	457-	169, 331	Epigallocatechin-3'-*O*-gallate	ND	5.29 ± 0.26	ND	5.29 ± 0.26
Procyanidin dimer (A2-type) [b]	4.13	575-	449, 423	Procyanidin dimer (B2-type)	20.98 ± 0.28	ND	ND	20.98 ± 0.28
Procyanidin dimer (B1-type) [a]	2.99	577-	425, 407	Procyanidin dimer (B1-type)	81.07 ± 3.81	2.32 ± 0.23	ND	83.39 ± 3.57
Procyanidin dimer (B2-type) [a]	3.46	577-	425, 407	Procyanidin dimer (B2-type)	129.32 ± 8.99	12.79 ± 0.68	ND	142.11 ± 9.69
Procyanidin trimer (A-type) [a]	3.32	863-	711, 411	Procyanidin trimer (A-type)	26.53 ± 1.74	ND	ND	26.53 ± 1.74
Procyanidin trimer (C1-type) [a]	3.09	865-	695, 739	Procyanidin trimer (C1-type)	43.26 ± 1.20	0.22 ± 0.01	ND	43.48 ± 1.22
Total Flavan-3-ols					**610.37 ± 68.76**	**167.32 ± 16.78**	**2.77 ± 0.63**	**780.46 ± 67.81**
**Flavanones**								
Hesperetin-7-*O*-rutinoside (hesperidin) [a]	3.84	609-	301, 285	Hesperetin-7-*O*-rutinoside	ND	1.59 ± 0.28	12.59 ± 0.32	14.17 ± 0.14
Naringenin-7-*O*-rutinoside [b]	3.69	579-	271, 151	Naringenin	ND	ND	5.91 ± 0.30	5.91 ± 0.30
Isosakuranetin-7-*O*-rutinosideoside [b]	4.39	593-	285, 270	Hesperetin-7-*O*-neohesperoside	ND	ND	16.55 ± 0.42	16.55 ± 0.42
Eriodictyol-7-*O*-rutinoside [b]	3.43	595-	287, 269	Hesperetin-7-*O*-rutinoside	ND	ND	0.25 ± 0.01	0.25 ± 0.01
Naringenin-4'-*O*-glucoside [b]	3.10	433-	271, 301	Hesperetin-7-*O*-neohesperoside	ND	ND	5.28 ± 0.24	5.28 ± 0.24
Hesperidin-3'-*O*-glucoside [b]	3.28	771-	463, 301	Hesperetin-7-*O*-neohesperoside	ND	ND	2.27 ± 0.25	2.27 ± 0.25
Hesperetin-7-*O*-neohesperoside-3'-*O*-glucoside [b]	2.77	771-	609, 463	Hesperetin-7-*O*-neohesperoside	ND	ND	2.24 ± 0.20	2.24 ± 0.20

Total Flavanones					**ND**	**1.59 ± 0.60**	**45.09 ± 5.99**	**46.68 ± 6.28**
**Flavones**								
Apigenin-6,8-C-diglucoside [b]	3.02	593^-	473, 503	Luteolin-4-glucoside	5.17 ± 0.68	1.63 ± 0.17	3.20 ± 0.11	10.00 ± 0.86
Total Flavones					**5.17 ± 0.68**	**1.63 ± 0.17**	**3.20 ± 0.11**	**10.00 ± 0.86**
**Flavonols**								
Quercetin [a]	4.56	301^-	179, 151	Quercetin	4.88 ± 0.57	ND	0.04 ± 0.01	4.92 ± 0.57
Myricetin-hexoside [b]	3 .30	479^-	317, 316	Luteolin-glucoside	ND	ND	0.31 ± 0.04	0.31 ± 0.04
Quercetin-3-glucoside [b]	3.54	463^-	301, 300	Quercetin-3-rutinoside	13.24 ± 0.91	2.79 ± 0.02	0.35 ± 0.00	16.37 ± 0.93
Dihydrokaempferol-hexoside [b]	3.15	449^-	287, 269	Luteolin-glucoside	ND	ND	0.72 ± 0.07	0.72 ± 0.07
Kaempferol-hexoside [b]	2.77	447^-	285, 286	Luteolin-glucoside	ND	ND	0.89 ± 0.07	0.89 ± 0.07
Syringetin-hexoside (isomer 1) [b]	3.28	507^-	327, 315	Procyanidin dimer (B1-type)	ND	ND	0.33 ± 0.05	0.33 ± 0.05
Syringetin-hexoside (isomer 2) [b]	3.52	507^-	345, 323	Procyanidin dimer (B1-type)	ND	ND	0.91 ± 0.01	0.91 ± 0.01
Quercetin-pentoside [b]	3.70	433^-	301, 300	Quercetin-3-rutinoside	13.52 ± 0.43	ND	ND	13.52 ± 0.43
Quercetin-3-rutinoside [a]	3.42	609^-	301, 300	Quercetin-3-rutinoside	ND	5.12 ± 0.13	ND	5.12 ± 0.13
Kaempferol-rutinoside [b]	4.39	593^-	285, 547	Luteolin-glucoside	ND	0.21 ± 0.04	ND	0.21 ± 0.04
Total Flavonols					**31.64 ± 5.59**	**8.12 ± 1.75**	**3.54 ± 0.37**	**43.29 ± 5.93**
**Anthocyanins**								
Delphinidin-3',5'-diglucoside [b]	2.27	627^+	465, 303	Delphinidin-3'-glucoside	ND	ND	1.57 ± 0.11	1.57 ± 0.11
Cyanidin-3',5'-diglucoside [b]	2.44	611^+	449, 287, 177	Cyanidin-3'-glucoside	ND	ND	11.01 ± 0.46	11.01 ± 0.46
Pelargonidin-3',5'-diglucoside [b]	2.61	595^+	433, 271	Pelargonidin-3'-glucoside	ND	ND	0.36 ± 0.01	0.36 ± 0.01
Delphinidin-3'-glucoside [a]	2.61	465^+	303, 305, 395	Delphinidin-3'-glucoside	ND	ND	1.71 ± 0.29	1.71 ± 0.29
Cyanidin-3'-glucoside [a]	2.77	449^+	287, 255, 269	Cyanidin-3'-glucoside	ND	ND	13.90 ± 0.72	13.90 ± 0.72
Pelargonidin-3'-glucoside [a]	2.80	433^+	271, 273, 350	Pelargonidin-3'-glucoside	ND	ND	0.64 ± 0.06	0.64 ± 0.06
Peonidin-3'-glucoside [a]	2.95	463^+	301, 395	Peonidin-3'-glucoside	ND	ND	0.78 ± 0.02	0.78 ± 0.02
Malvidin-3'-glucoside [a]	2.99	493^+	331, 279	Malvidin-3'-glucoside	ND	ND	0.85 ± 0.06	0.85 ± 0.06
Cyanidin-3'-rutinoside [a]	2.80	595^+	120, 316	Cyanidin-3'-rutinoside	ND	ND	0.42 ± 0.04	0.42 ± 0.04
Total Anthocyanins					**ND**	**ND**	**31.24 ± 5.16**	**31.24 ± 5.16**
**Total phenolic compounds**								
					**647.17 ± 24.79**	**191.60 ± 5.40**	**387.00 ± 20.46**	**1226 ± 40**

RT: Retention time; [a]: Compounds identified by comparing retention times and MS data with those of reference compounds; [b]: compounds (tentatively) identified; ND: Not detected.
